# All‐Printed Finger‐Inspired Tactile Sensor Array for Microscale Texture Detection and 3D Reconstruction

**DOI:** 10.1002/advs.202400479

**Published:** 2024-05-02

**Authors:** Yilin Wang, Jiafeng Zhao, Xu Zeng, Jingwen Huang, Yading Wen, Juergen Brugger, Xiaosheng Zhang

**Affiliations:** ^1^ School of Integrated Circuit Science and Engineering University of Electronic Science and Technology of China Chengdu 611731 China; ^2^ Microsystems Laboratory Ecole Polytechnique Fédérale de Lausanne Lausanne 1015 Switzerland

**Keywords:** all‐printed, bio‐inspired, braille recognitions, human‐machine interfaces, tactile sensors, texture detections, texture reconstructions

## Abstract

Electronic skins are expected to replicate a human‐like tactile sense, which significantly detects surface information, including geometry, material, and temperature. Although most texture features can be sensed in the horizontal direction, the lack of effective approaches for detecting vertical properties limits the development of artificial skin based on tactile sensors. In this study, an all‐printed finger‐inspired tactile sensor array is developed to realize the 3D detection and reconstruction of microscale structures. A beam structure with a suspended multilayer membrane is proposed, and a tactile sensor array of 12 units arranged in a dual‐column layout is developed. This architecture enables the tactile sensor array to obtain comprehensive geometric information of micro‐textures, including 3D morphology and clearance characteristics, and optimizes the 3D reconstruction patterns by self‐calibration. Moreover, an innovative screen‐printing technology incorporating multilayer printing and sacrificial‐layer techniques is adopted to print the entire device. In additon, a Braille recognition system utilizing this tactile sensor array is developed to interpret Shakespeare's quotes printed in Grade 2 Braille. The abovementioned demonstrations reveal an attractive future vision for endowing bioinspired robots with the unique capability of touching and feeling the microscale real world and reconstructing it in the cyber world.

## Graphical abstract

A finger‐inspired tactile sensor array comprising of a microbeam fabricated via screen printing is proposed. Such a tactile sensor array has been demonstrated to detect microscale textures in both the horizontal and vertical domains, realizing 3D detection and reconstruction of microscale structures. This opens a chapter for intelligent robotic fingers moving forward to the micro‐spatial world.

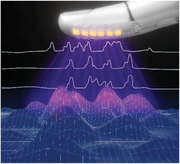

## 1 Introduction

The skin is a natural and precise multifunctional sensor that enables humans to detect features of their surroundings. Numerous researchers have focused on studying the skin and mimicking it to create electronic skins (E‐skins) that match and surpass the sensing ability of human skin. Currently, E‐skins possess the ability to detect various factors such as pressure,^[^ ^1^, ^2^, ^3^, ^4^ ^]^ temperature,^[^ ^5^, ^6^, ^7^, ^8^ ^]^ humidity,^[^ ^9^, ^10^, ^11^, ^12^ ^]^ and biochemical characteristics.^[^ ^13^, ^14^, ^15^, ^16^ ^]^ These advancements hold significant promise for applications in intelligent robotics,^[^ ^17^, ^18^, ^19^, ^20^ ^]^ health care,^[^ ^21^, ^22^, ^23^, ^24^ ^]^ prosthetics,^[^ ^25^, ^26^, ^27^, ^28^ ^]^ and human‐machine interfaces.^[^ ^29^, ^30^, ^31^, ^32^ ^]^ However, despite notable progress, achieving accurate tactile perception similar to human skin, particularly in detecting and reconstructing the texture features of touched objects, remains a significant challenge.

The fundamental aspect of tactile detection is its sensitivity to diverse mechanical stimuli from the surroundings, such as pressure, bending, tapping, slipping, and vibration. Tactile sensors have been developed to convert mechanical stimuli into electrical signals by employing different physical transduction mechanisms, including piezoresistivity,^[^ ^33^, ^34^, ^35^, ^36^ ^]^ capacitance,^[^ ^37^, ^38^, ^39^, ^40^ ^]^ piezoelectricity,^[^ ^41^, ^42^, ^43^, ^44^ ^]^ triboelectricity,^[^ ^45^, ^46^, ^47^, ^48^ ^]^ acoustics,^[^ ^49^, ^50^, ^51^ ^]^ optics,^[^ ^52^, ^53^, ^54^ ^]^ and magnetism.^[^ ^55^, ^56^, ^57^ ^]^ Because the sensitivity of a tactile sensor is pivotal for accurate texture detection, researchers have sought to enhance it by designing various microstructures, such as pillars,^[^ ^58^, ^59^, ^60^, ^61^ ^]^ pyramids,^[^ ^62^, ^63^, ^64^, ^65^ ^]^ and hemispheres.^[^ ^66^, ^67^, ^68^, ^69^ ^]^ However, most of the tactile sensors that have been proposed can only detect texture features in the horizontal direction. The only features in the vertical direction that can be detected are the two states of high or low; however, it is impossible for these sensors to perceive “how high” the texture is.

**Figure 1:**
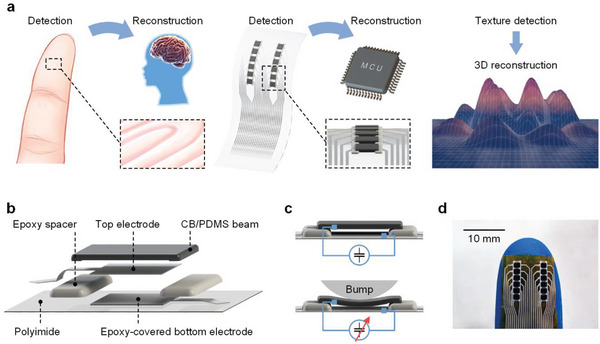
Concept, structure, and sensing mechanism of the tactile sensor array. a) Inspired by the behavior of finger tactile sense, that is, the detection‐reconstruction capability for 3D microscale textures, an all‐printed tactile sensor array (TSA) is proposed. In contrast with traditional tactile sensors, the developed TSA is highly sensitive in both horizontal and vertical domains to capture the true 3D information and reconstruct it as a 3D model. b) The structure diagram of the sensing unit composed of a beam, spacers, electrodes, and flexible substrate to form a unique multilayer suspended configuration, an optimal screen‐printing technology is adopted to print suspended structures layer‐by‐layer precisely. c) Schematic diagram of the sensing principle. d) Photograph of the TSA attached to the finger.

Human tactile sense is a 3D spatial perception behavior. When a finger touches the surface of an object, it can detect not only the contour of the texture in the horizontal direction but also its height characteristics in the vertical direction. Subsequently, the brain reconstructs a 3D model of the texture. Herein, we propose a tactile sensor array (TSA) mimicking the behavior of human tactile perception to detect the 3D morphological features of textures and incorporate an electronic system to achieve the 3D reconstruction of texture patterns, as shown in **Figure** **1**a. The proposed TSA consists of two columns of sensing units with beam structures sensitive to microbumps like fingerprints. The dual‐column layout of the TSA enables it to detect interval characteristics, such as the distance information in a texture, and optimize the reconstructed texture patterns. Moreover, we implement a Braille recognition system based on the proposed sensor array.

It is worth mentioning that, there are several existing manufacturing technologies for flexible sensors with their own characteristics. For example, additive manufacturing technology can process complex 3D structures,^[^ ^70^ ^]^ photolithography has very high processing accuracy,^[^ ^71^ ^]^ and roll‐to‐roll processing is fast to produce.^[^ ^72^ ^]^ We chose screen printing to complete the entire processing of the proposed sensor array for its high processing accuracy, flexibility in material selection, mass‐fabrication and cost‐effectiveness. Moreover, the sacrificial layer technology was introduced into screen printing to realize a 3D beam structure by screen printing. This all‐printed tactile sensor array shows the potential of highly integrated printed electronics for future applications in electronic skins, human‐machine interfaces, and intelligent robots.

## 2 Results and Discussion

### 2.1 Design of the Beam‐Structure TSA

Figure 1a shows the behavior of human tactile senses. The fingers are sensitive to microscale 3D structures, and the brain reconstructs a 3D model of the touched objects after receiving the corresponding nerve signals. To mimic this behavior, we designed a finger‐inspired TSA to detect microscale textures in both the horizontal and vertical domains. We also designed an electronic system to reconstruct textures in 3D directions, just like the brain. The TSA consists of two sensor columns, each comprising six sensing units. Figure 1b schematically shows the structure of the sensing unit consisting of a polyimide (PI) substrate, epoxy‐covered silver bottom electrode, epoxy spacers, silver top electrode, and a carbon black/polydimethylsiloxane (CB/PDMS) beam. The CB/PDMS beam is soft and easily deformed, acting as the sensitive part, whereas the epoxy spacers are sufficiently rigid to keep the beam suspended. Owing to the beam structure, the proposed sensing unit is highly sensitive to pressure from the microbumps. The sensing mechanism is shown in Figure 1c. When bumps deform the beam, the capacitance of the unit increases owing to a decrease in the gap between the electrodes. The deformation process is shown in Video S1 (Supporting Information). Because of the individual beams of each sensing unit, there is no signal crosstalk between the units, as shown in Figure S1 (Supporting Information). The sensor array is processed on a 25 µm‐thick PI film by screen‐printing technology; thus, the device is highly flexible to adhere to the finger (Figure 1d).

**Figure 2:**
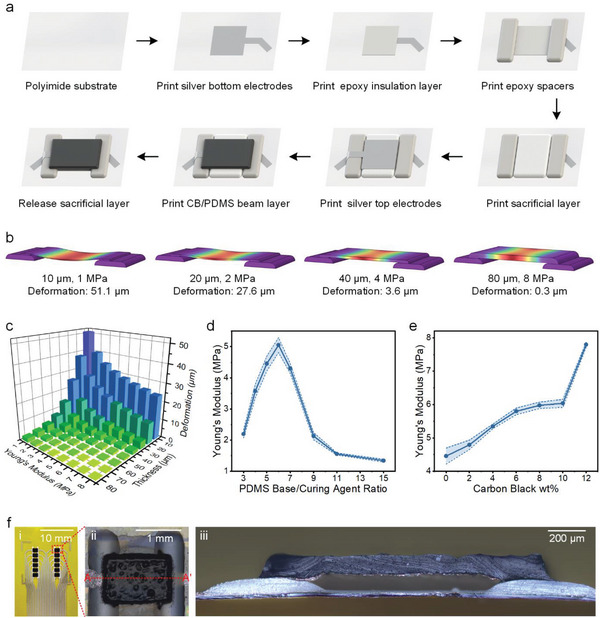
Fabrication, simulation, and morphology characterizations of the TSA. a) The fabrication flow chart of the TSA using screen‐printing technology combining multilayer printing and sacrificial‐layer technology. b,c) Simulation results of the deformation of beams with various layer thicknesses and Young's modulus values. d) Young's modulus of the PDMS films with different PDMS base‐to‐curing agent ratios. e) Young's modulus of CB/PDMS (the ratio of the PDMS base to the curing agent is 5:1) films with different weight percentages of carbon black. f) Photographs of the (i) TSA, (ii) sensing unit, and (iii) cross‐section of a sensing unit.

The sensor array is entirely fabricated by screen‐printing technology. Thus, all the units can be fabricated simultaneously. The fabrication flow chart of a single unit is shown in **Figure** **2**a to illustrate the fabrication process of the sensor array. First, bottom electrodes and wires (width: 300 µm) are printed on PI film with silver paste. An epoxy layer is printed onto the electrodes for insulation. Owing to its high heat distortion temperature, epoxy is selected as the insulation and spacer material. The spacers are then overprinted layer‐by‐layer to achieve the desired thickness. We overprinted the spacers 6 times to a thickness of 80 µm. To fabricate the suspended beam, a sacrificial layer is overprinted between the spacers several times to achieve the same thickness as the spacers, providing temporary support. A trimethylolethane (TME) / trimethylolpropane (TMP) composite is selected as the sacrificial material, which sublimes at 150 °C.^[^ ^73^ ^]^ Subsequently, electrodes are printed on the top of the sacrificial layer. The top layer is the beam, which acts as a movable part. PDMS is selected as the beam material because of its flexibility. Last, the sacrificial layer is released by heating the device to 150 °C for 30 min.

We noticed that if the thickness or stiffness of the beam is inadequate, it will sag after the release of the sacrificial layer. We obtained devices with different beam thicknesses by controlling the number of overprints; devices with fewer than five overprints exhibited sagging beams (Figure S2, Supporting Information). To investigate the effect of Young's modulus of the material and layer thickness on the deformation of the beam, a model of the sensing unit is constructed in COMSOL. The deformation of the beam is simulated by varying Young's moduli and thicknesses. Model examples of the simulation are shown in Figure 2b, and the simulation results for the maximum deformation of the beam are plotted in Figure 2c. During actual processing, the deformation of the beam layer within 5 µm will not affect the overall structure. Therefore, we selected the appropriate layer thickness and Young's modulus from the simulation results. To obtain good mechanical properties, the layer thickness is designed to be between 60 and 100 µm, and Young's modulus is designed to be between 3 and 8 MPa. On the one hand, the layer thickness can be controlled by adjusting the number of overprints. On the other hand, Young's modulus can be controlled by adjusting the PDMS base‐to‐curing‐agent ratio and doping carbon black in the PDMS matrix. We measured the stress‐strain curves of the PDMS films with different PDMS base‐to‐curing‐agent ratios and CB/PDMS films (the ratio of the PDMS base to curing agent is 5:1) with varying percentages of weight of carbon black (Figures S3 and S4, Supporting Information). We calculated their Young's modului, as shown in Figure 2d,e. Collectively, these results provide important guidelines for material preparation and device processing.

Figure 2f shows photographs of (i) the sensor array, (ii) the details of the sensing unit, and (iii) the cross section at A‐A’ in (ii). The beam size is 1.5 mm × 1 mm × 0.1 mm (length × width × thickness). The spacing between neighboring units in the same column is 0.5 mm, and the distance between the columns is 6.6 mm. The height of the cavity and spacers is ≈ 80 µm, and the width of the cavity (i.e., the spacing between spacers) is 1 mm. Photographs labeled with dimensional information are shown in Figure S5 (Supporting Information).

### 2.2 Characterization of the Sensing Unit for Texture Height Detection

**Figure 3:**
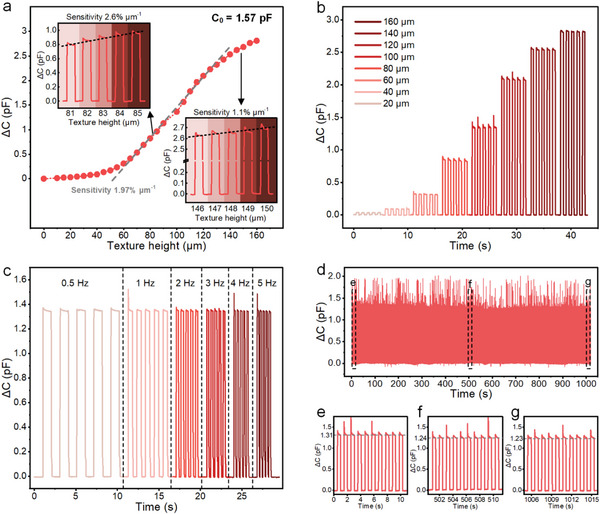
Sensing performance of the sensing unit for texture height detection. a) Relative capacitance changes of the sensing unit at different texture heights. The inset in the upper left corner shows the capacitive responses of the sensing unit to different texture heights from 81 to 85 µm, where the sensing unit has a sensitivity of 2.6% µm^−1^. The inset in the lower right corner shows the capacitive responses of the sensing unit to different texture heights from 146 to 150 µm, where the sensing unit has a sensitivity of 1.1% µm^−1^. b) The capacitive responses of the sensing unit when detecting textures with a height in the range of 20–160 µm with a detecting frequency of 1 Hz. c) Capacitive response of the sensing unit for periodically detecting a 100‐µm height at different frequencies. d) Cycling stability of the sensing unit at an applied height of 100 µm with a frequency of 1 Hz. e–g) The beginning, intermediate, and final cycles.

According to the beam deformation sensing mechanism shown in Figure 1c, the fabricated sensing unit is susceptible to texture height. To characterize the effectiveness of the sensor's response to different texture heights, we prepared a customized testbench composed of a precision electric moving stage, an LCR meter, and a 3D‐printed bump, as shown in Figure S6 (Supporting Information), Furthermore, we used the distance the cavity is compressed to represent the height of the texture that the sensor touches. **Figure** **3**a shows the capacitance changes (ΔC) of the sensing unit at different texture heights. The initial capacitance of the sensing unit (C_0_) is 1.57 pF, and the ΔC is 2.81 pF when the texture height increases to 160 µm. The sensor is extremely sensitive when the texture height is 70–125 µm, with an overall sensitivity of 1.9% µm^−1^ in this range. We measured the sensitivity of the sensor at texture heights of ≈ 85 and 150 µm, where the sensitivity reaches 2.6 and 1.1% µm^−1^, respectively. Figure 3b shows the response curve of the sensing unit for detecting heights in the range of 20–160 µm with a detection frequency of 1 Hz. The response curve of the sensing unit for detecting a height of 100 µm at different frequencies is shown in Figure 3c. When the detection frequency changes from 0.5 to 5 Hz, the peak ΔC remains constant, which indicates that the touch speed does not affect the height detection result. The stability of the sensing unit is illustrated in Figure 3d–g. During the 1000‐cycle detection process at 100 µm with a frequency of 1 Hz, the amplitude of ΔC declined relatively high in the first 500 cycles but kept almost consistent later in the following 500 cycles.

**Figure 4:**
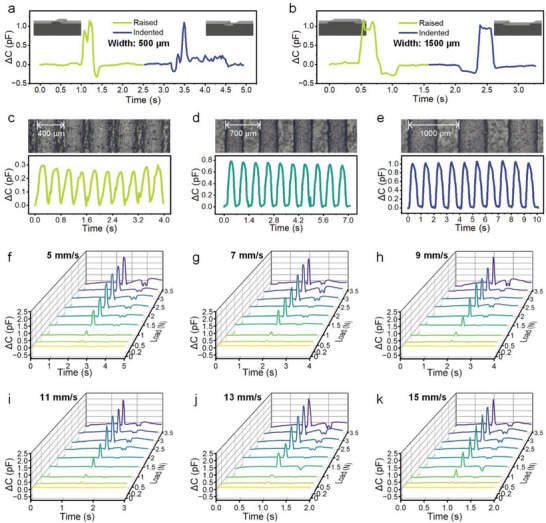
Sensing performance of the TSA during scanning. a) Comparison of the ΔC change curves of the sensor when scanning over 100‐µm‐raised and 100‐µm‐indented bars with a width of 500 µm. b) Comparison of the ΔC change curves of the sensor when scanning over 100‐µm‐raised and 100‐µm‐indented bars with a width of 1500 µm. c–e) Response curves of the sensing unit when the TSA scans on periodic bars with spatial periods of 400, 700, and 1000 µm, respectively. f–k) Response curves of the sensing unit when the TSA scans over a 500‐µm‐wide and 100‐µm‐high bar with different loads and scanning speeds.

In subsequent studies, texture features were detected by sensor scanning; therefore, the sensing performance of the moving sensor was also evaluated, as shown in **Figure** **4**. Similar to a human finger, the proposed sensor can distinguish between raised and indented structures in the scanning path, as reflected in the response curve. Figure 4a shows the response curves of the sensor when scanning over 100‐µm‐raised and 100‐µm‐indented strips that are both 500 µm in width, whereas the width of the structures in Figure 4b is 1500 µm. If the raised height is equal to the indented depth, the amplitudes of response curves are approximate. But, the waveforms are mirror‐reflected, there is a positive pulse followed by a negative pulse in the response curve when scanning raised structures, whereas there is a negative pulse followed by a positive pulse in the response curve when scanning indented structures. Benefiting from the beam structure, the sensor could discern textures with microspacing during scanning. Periodic bars with spatial periods of 400, 700, and 1000 µm were prepared using a 3D printer respectively. As the TSA scans over the bars, the response curve exhibits a corresponding periodicity, as shown in Figure 4c–e. The speed of the scanning TSA is 1 mm s^−1^, and its product with the period of the waveform is exact spatial period of the bars.

In addition, the effects of load and speed on the output signal during scanning were investigated. During the experiment, a difference in the load was achieved by varying the mass of the weights placed on the TSA. With different loads and scanning speeds, the TSA scanned over a 500‐µm‐wide and 100‐µm‐high bar, and the capacitance variation of one sensing unit in the front column was measured. The results are shown in Figure 4i–n. In these waveforms, the positive pulses are generated when the unit slides over the bar. In contrast, negative pulses result from the vibration of the device as the rear sensing units slide over the bar. When the load is below 0.2 N, the signal is virtually submerged in noise. In contrast, when the load exceeds 3 N, the vibration of the device becomes increasingly pronounced, leading to more severe signal distortion. Overall, loads ranging from 1 to 2.5 N are suitable. In addition, scanning speeds below 15 mm s^−1^ do not significantly affect the test results, and the upper limit of the scanning speed is contingent on the sampling rate of the capacitance measurement circuit.

### 2.3 Distance Detection Capability of the TSA

**Figure 5:**
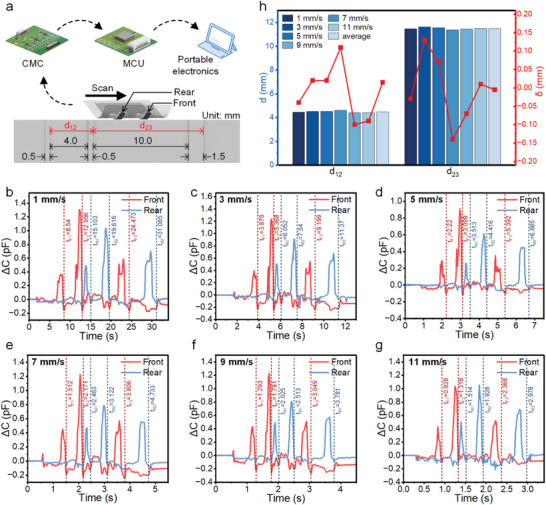
Sensing performance of the TSA for texture distance detection. a) Schematic diagram of the sensor‐array‐capacitance measurement method for realizing texture distance detection during sensor array scanning. b–g) The capacitive response of the front and rear sensing units while the sensor array scans the texture with different velocities. t_Fn_ and t_Rn_ represent the times to reach the lowest points of the falling edge of the nth pulse in the output signals of the front and rear units, respectively. h) The calculated values and errors of *d*_12_ and *d*_23_ at different scanning velocities.

The dual‐column layout imparts distance‐detection capabilities to the sensor array. When the sensor array scans a texture with uniform rectilinear motion along a path parallel to the rows, a fixed phase difference exists between the two units within the same row, owing to their constant relative positions. This phase difference is inversely proportional to the speed of the sensor array. By utilizing the known distance between these two units and measuring the phase difference, the sensor array's speed can be calculated, thus enabling the characterization of the distance of the texture. An experiment was conducted to demonstrate the distance detection capability of the TSA, as illustrated in **Figure** **5**a. During the experiment, the sensor array scanned the surface of a 3D‐printed plate from left to right, where three bars were located on the plate.

To obtain the real‐time capacitance of each unit, we designed a capacitance measurement circuit (CMC) consisting of three capacitance‐to‐digital converters and 12 measurement channels. In addition, a microcontroller unit (MCU) controls the converters and transfers data to portable electronics. Figure 5b–g show the output signals from the two units in the same row during scanning at different velocities. In these signals, each pulse corresponds to a scanned bar. The times required to reach the lowest points of the falling edge of the nth pulse in the output signals of the front and rear units are defined as *t*_Fn_ and *t*_Rn_, respectively. The average phase difference between the output signals of the front and rear units (${\bar{\Delta t}}_{FR}$) can be calculated according to Equation (1).

(1) $${\bar{\Delta t}}_{FR}=\frac{t_{R1}-t_{F1}+t_{R2}-t_{F2}+t_{R3}-t_{F3}}{3}$$

The scanning speed (*v*) of the sensor array can be calculated according to Equation (2) as follows:

(2) $$v\;=\frac{d_{FR}}{{\bar{\Delta t}}_{FR}}\;$$

where *d_FR_* is the distance between the front and rear units and is equal to 6.6 mm. Subsequently, the distance between the right edges of the first and second bars *d*_12_ and the distance between the right edges of the second and third bars *d*_23_ can be calculated using the Equations (3) and (4) as follows:

(3) $$\begin{array}{ccc} d_{12} & = & {\bar{\Delta t}}_{12}\;\times v\;=\frac{t_{R2}-t_{R1}+t_{F2}-t_{F1}}{2}\;\times \;\frac{d_{FR}}{{\bar{\Delta t}}_{FR}}\\ & = & \frac{3d_{FR}\left(t_{R2}-t_{R1}+t_{F2}-t_{F1}\right)}{2\left(t_{R1}-t_{F1}+t_{R2}-t_{F2}+t_{R3}-t_{F3}\right)}\; \end{array}$$

(4) $$\begin{array}{ccc} d_{23} & = & {\bar{\Delta t}}_{23}\;\times v\;=\frac{t_{R3}-t_{R2}+t_{F3}-t_{F2}}{2}\;\times \;\frac{d_{FR}}{{\bar{\Delta t}}_{FR}}\\ & = & \frac{3d_{FR}\left(t_{R3}-t_{R2}+t_{F3}-t_{F2}\right)}{2\left(t_{R1}-t_{F1}+t_{R2}-t_{F2}+t_{R3}-t_{F3}\right)}\; \end{array}$$

The actual values of *d*_12_ and *d*_23_ are 4.5 mm (4 mm + 0.5 mm) and 11.5 mm (10 mm + 1.5 mm), respectively. The calculated values and errors of *d*_12_ and *d*_23_ at different scanning velocities are presented in **Table** **1** and Figure 5h, respectively.

**Table 1:** Calculated values and errors of ***d***_12_ and ***d***_23_ at different scanning velocities.

	*d*_12_ [mm]		*d*_23_ [mm]	
	Result	Error	Result	Error
1 mm s^−1^	4.46	−0.04	11.47	−0.03
3 mm s^−1^	4.52	0.02	11.63	0.13
5 mm s^−1^	4.52	0.02	11.57	0.07
7 mm s^−1^	4.61	0.11	11.36	−0.14
9 mm s^−1^	4.40	−0.1	11.43	−0.07
11 mm s^−1^	4.41	−0.09	11.51	0.01
Average	4.487	0.015	11.495	−0.005

The results indicate that in the proposed distance detection method, the scanning speed has no effect on the detection results, and the error in the one‐time detection is less than 0.15 mm. Moreover, the error in the average result of the 6‐time detection is less than 0.015 mm. The accuracy of the distance detection can be further improved by increasing the smoothness of the scanning process and the sampling rate of the CMC.

### 2.4 Pattern Reconstruction and Optimization

The sensitivity of TSA to the texture height enables the detection of patterns formed by surface bumps. The pattern is reconstructed by plotting a time‐dependent intensity graph of the output signals from the sensing units in one column.

**Figure 6:**
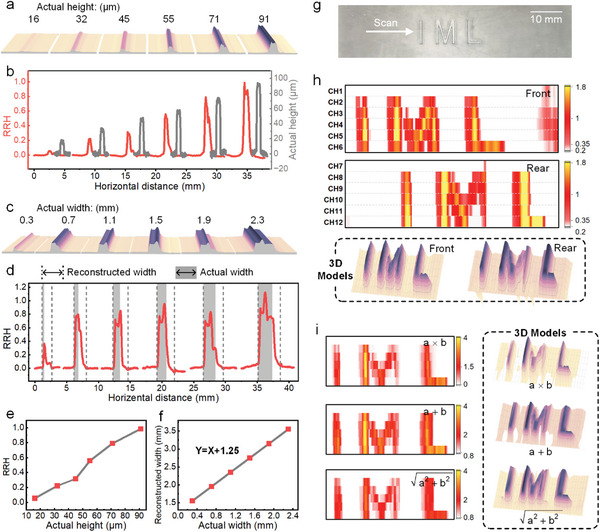
Pattern reconstruction and optimization capabilities of the TSA. a) Reconstructed models of bars of different heights by a sensing unit of the TSA. b) The comparison between reconstructed relative heights (RRH) and actual heights of these bars. c) Reconstructed models of bars of different widths by a sensing unit of the TSA. d) The dimensional information of the cross‐section of the models. The width intercepted by a pair of dashed lines is the width of the reconstructed model, and the width shaded in grey is the width of the real structure. e) The quantitative relationship between reconstructed relative height and actual height. f) The quantitative relationship between reconstructed width and actual width. g) Photograph of a 3D‐printed surface pattern in the shape of the word “IML”. h) 2D images and 3D models of the pattern reconstructed by the front and rear column sensors, respectively. CH1‐CH6 are in the front of the sensor array, whereas CH7‐CH12 are in the rear. i) Optimized 2D images and corresponding 3D models of the pattern obtained by coupling the sensing results of dual‐column sensor units with different algorithms.

The performance of TSA in reconstructing textures of different widths and heights is evaluated, as shown in **Figure** **6**a–f. Figure 6a shows reconstructed models of bars of different heights by a sensing unit of the TSA, and the stylus profiler measured the heights of actual structures to be 16, 32, 45, 55, 71, and 91 µm. The reconstructed relative heights (RRH) and actual heights of these bars are compared in Figure 6b.e, which indicate that the RRH monotonically increases with the actual height. Therefore, the data from the TSA can reconstruct models with relative heights to show the difference in the heights of the textures. In the future, a library of mappings from ΔC to actual heights can be built based on a large amount of test data, allowing the reconstruction of models with accurate heights.

To evaluate the influence of the width of the texture on the reconstruction, the TSA scanned over the 3D printed bars with the same height but different widths one by one, and their actual widths were measured under a microscope to yield 0.3, 0.7, 1.1, 1.5, 1.9, and 2.3 mm, respectively, as shown in Figure S7 (Supporting Information). Figure 6c shows the reconstructed model from the sensing unit, and the dimensional information of the cross‐section of the model is shown in Figure 6d. In Figure 6d, the width intercepted by a pair of dashed lines is the width of the reconstructed model, and the width shaded in gray is the width of the real structure. The reconstructed width was always a fixed value larger than the actual width, as shown in Figure 6f. Consequently, the width information of the actual structure can be effectively calculated from the reconstructed results. The fixed value is 1.25 mm, which coincides with the length of the movable part of the beam. From the time when the beam touches the bar and deforms to the time when the beam leaves the bar and recovers, the sensor moves a distance equal to the width of the bar plus the length of the beam. In addition, from the models and curves, when the width of the bar is 0.3 mm, its RRH is significantly smaller than that of other widths. This is because the sensitive part of the sensor is a soft CB/PDMS beam, and only the part of the beam that touches the bump deforms. If the bump is smaller than the beam, only a part of the top electrode approaches the bottom electrode along the beam; thus, the capacitance change of the sensor is smaller.

A 3D‐printed surface pattern in the shape of the word “IML” is shown in Figure 6g. When TSA scans the surface texture, the capacitance of each sensing unit varies according to the height of the texture in the path, as shown in Figure S8 (Supporting Information). The signal from each channel was plotted as an intensity graph, and 2D images of the reconstructed patterns were obtained by stitching the intensity graphs of CH1‐CH6 or CH7‐CH12. CH1‐CH6 refer to the signals from the sensing units in the front column, and CH7‐CH12 refer to the signals from the sensing units in the rear column. On this basis, 3D models of the patterns can be obtained, as shown in Figure 6h. These two models roughly reconstruct the shape of the pattern shown in Figure 6g.

The reconstructed models were incomplete, and the surface was rough because the scanning process was performed manually (Video S2, Supporting Information). However, the model could be optimized using the self‐calibration ability of the TSA. By leveraging the dual‐column layout, a single scan yields two data sets on the patterns features. Algorithmic operations on the two sets of data can reduce the error and lead to optimization of the reconstructed patterns. Specifically, this is accomplished by shifting the two 2D images such that the patterns essentially overlap and then operating on the values of the pixels in the same position in both 2D images. The three optimization algorithms used were sum (a + b), product (a × b), and L2‐norm ($\sqrt{a^{2}+b^{2}}$). The optimized 2D images and the corresponding 3D models are shown in Figure 6i. The optimized 3D model is more regular and closer to the real shape. To evaluate the reconstructed model, the height profiles of the actual structure were measured using a stylus profiler and compared with those of the reconstructed model optimized using the L2‐norm. There is a high degree of detailed similarity between the model and the actual structure, as shown in Figure S9 (Supporting Information). Therefore, it was demonstrated that a dual‐column layout could not only reconstruct texture patterns but also optimize the reconstructed models.

### 2.5 TSA‐Based Braille Recognition System

**Figure 7:**
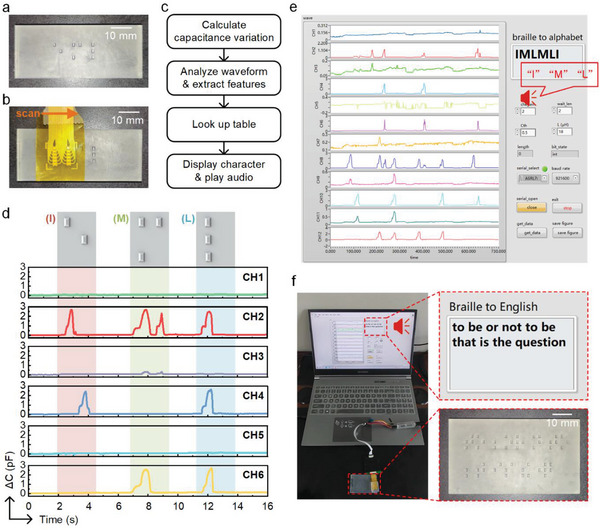
TSA‐based Braille recognition system. a) 3D‐printed Braille characters including “I”, “M” and “L”. b) Illustration of Braille recognition by scanning on Braille characters with the TSA. c) Flow chart of the computer program for Braille recognition. d) The Braille characters corresponding to “I”, “M”, and “L”, and the capacitive responses of sensing units. CH1 to CH6 are in the same column in the sensor array. The capacitance of the unit will increase when the unit reaches the Braille dots. e) The front panel of the recognition program after the Braille characters are recognized successfully. The computer will read out each alphabet that has been recognized. f) Photograph of recognizing a Grade 2 Braille sentence. The computer shows and reads the recognition results while the TSA scans over the Braille plate.

Each Braille character comprises one to six Braille dots that can be detected by the proposed TSA and further recognized in conjunction with a program. In this study, we designed a Braille recognition system composed of the TSA, the CMC, the MCU, and the portable electronics running the recognition program, and the system block diagram is the same as that shown in Figure 5a. **Figure** **7**a shows a 3D‐printed plate with three Braille dotted characters; its dimensions are shown in Figure S10 (Supporting Information). These Braille characters correspond to “I”, “M”, and “L” respectively. When the TSA scans over a Braille character (Figure 7b), the sensing units in contact with the dots deform and thus exhibit a changing capacitance. CMC measures the capacitance and converts it into digital signals. The signal is then transferred to the host computer (e.g., portable electronics), and a program analyzes the data from all channels in real time. A flow chart of the Braille recognition program is illustrated in Figure 7c. After receiving data from the MCU through a serial port, the program calculates the value and variation of the capacitance of each channel. It analyzes the waveform of the capacitance variation in the time domain to extract the features of each Braille character. The corresponding letter is then found in the look‐up table based on the extracted features. Finally, the recognition result is shown, and the alphabet audio is played simultaneously. The proposed method is achieved by recognizing the sequence of pulses in the signal; thus, the recognition results can be generated in real time, and the recognition of an arbitrary Braille can be achieved by simply adding the sequence of that Braille dots in the lookup table.

Braille recognition can be achieved using one TSA sensing column. Figure 7d illustrates signals from CH1 to CH6 (representing the capacitance variations of 6 sensing units in the same column) when the sensor array scans over the Braille characters referring to “I”, “M”, and “L”. Based on these signals, the program can distinguish the location of the blind dots and thus recognize the Braille characters. Figure 6e shows the front panel of the program after the Braille characters were recognized successfully, and a demonstration video is shown in Video S3 (Supporting Information). Furthermore, we printed the sentence “to be or not to be, that is the question” quoted from Shakespeare in Grade 2 Braille on a 3D‐printed plate. The program recognized the Braille and read it successfully using TSA scanning over the Braille. Figure 7f shows the recognition scene and sentence in Grade 2 Braille, and a demo video is shown in Video S4 (Supporting Information).

## 3 Conclusion

In this work, we introduced a 3D texture detection and reconstruction system that behaves similarly to the human tactile sense. This system is based on a novel tactile sensor array with a dual‐column layout comprising sensing units with beam structures. Our results demonstrate that the tactile sensor possesses a high sensitivity of 2.6% µm^−1^ to texture height, and the tactile sensor array can obtain the texture features and realize 3D reconstruction of complex texture patterns by scanning. The dual‐column layout was demonstrated to endow the tactile sensor array with texture distance detection ability, with an error of less than 0.015 mm in the average result of six detections. In addition, we employed three algorithms to couple the data from the two sensing columns to optimize the reconstructed models. Based on this tactile sensor array, we successfully implemented a real‐time Braille recognition system, showcasing its ability to recognize Braille characters. Despite the high performance of the proposed sensor array, it still exhibits some limitations such as low spatial resolution and strict motion status requirements. Further efforts should focus on miniaturizing the sensing unit and increasing their number to address the low spatial resolution. To meet the motion status requirements, the sensor array can be used in application scenarios such as prosthetics and robots to avoid the problem.

Furthermore, we provided a detailed description of the fabrication process of the TSA using screen printing technology. Our approach achieves the proposed 3D structure through multilayer printing and sacrificial‐layer technique within the confines of a planar process. In the future, through innovative screen‐printing technology, we aim to improve the sensor density to achieve higher spatial precision and integrate diverse sensing mechanisms to enhance multifunctionality.

Overall, our proposed tactile sensor array and fabrication process present a new strategy for the batch processing of high‐density flexible sensors. They have promising broad applications across various fields, particularly in electronic skins, intelligent robots, and human‐machine interfaces.

## 4 Experimental Section

### Preparation of the Sacrificial Material Paste

The sacrificial material paste was composed of trimethylolethane (HEOWNS), trimethylolpropane (RHAWN), cyclohexanol (RHAWN), and propylene glycol (MACKLIN) in a weight ratio of 6:1:4:1. First, a mixture of the four ingredients was heated and stirred on a thermostatic magnetic stirrer (B11‐2, Shanghai Sile Instruments Co., Ltd., China) at a temperature of 100 °C and a stirring speed of 300 rpm until the solids were completely dissolved. The solution was cooled to room temperature to obtain a paste suitable for screen printing.

### Preparation of the CB/PDMS Paste

To ensure that the PDMS paste had the right viscosity for screen printing, carbon black (K90, RHAWN) was mixed into PDMS (Sylgard 184, Dow Corning) at a curing agent to base ratio of 1:5. The weight percentage of carbon black, from 5% to 10%, met the requirements. The mixture was stirred for 20 min and placed in a vacuum chamber to remove the air bubbles.

### Measurement of Young's Modulus

The PDMS base and curing agent were mixed at different ratios in plastic containers. PDMS with a base‐to‐curing‐agent ratio of 5:1 was divided into several portions, to which carbon black of varying weight percentages was added and stirred thoroughly. These PDMS pastes and CB/PDMS pastes were placed in a vacuum chamber to remove air bubbles and cured in an oven at a temperature of 70 °C for 30 min. A vertically movable force gauge (HP‐100, HANDPI) with a position display was placed above the acrylic plate. The contact surface of the force gauge probe with the object was a 3 mm × 3 mm square. First, the force gauge was moved to the position where it touched the acrylic plate, and this position was noted as the origin. The force gauge was then raised, and a cured film was attached to the acrylic board. The force gauge was lowered again to ensure that it just touched the film, and the position of the force gauge at this point was the original thickness of the film. The force gauge was then slowly lowered, and the position of the force gauge and the magnitude of the force were recorded. This way, the stress can be calculated according to σ = *F/A*, where σ is the stress, *F* is the force of compression, and *A* is the cross‐sectional surface area. The strain can be calculated according to ε = Δ*L/L_0_*, where ε is the strain, Δ*L* is the change in length, and *L_0_* is the original length (i.e., the original thickness of the film). Finally, a linear fit was made to the data of (σ, ε), and the slope of the fitted line is the Young's modulus of the film.

### Fabrication of the Tactile Sensor Array

The tactile sensor array (TSA) was fabricated by screen printing using a screen printer (QY‐HP‐2525HPB, Shanghai Hoting Screen Printing Equipment Co., Ltd., China) and customized 300‐mesh stencils. Silver electrodes and wires were printed using commercially available silver ink (SharEx Co., Ltd., China). The curing temperatures of the bottom and top electrodes are 150 and 70 °C, respectively, because a high curing temperature of the top electrodes above 80 °C will lead to the sublimation of sacrificial materials. The insulation layer and spacers were printed using epoxy screen‐printing ink (Huizhou Saide Industrial Materials Co., Ltd, China) at a curing temperature of 150 °C. During the overprinting of the spacers, a curing process at 150 °C for 10 min was required after each printing. Similarly, curing processes at 70 °C for 10 min after each printing were also necessary during the overprinting of sacrificial layers and beams.

### Characterization and Measurements

All 3D printed molds for testing were processed using a 3D printer (Form 3+, Formlabs), and a stylus profiler (Dektak 150, Bruker) was used to measure their profiles.

The texture height detection performance of the sensing units was characterized using a precision electric moving stage (DZ425TA200S, Chengdu Dingyou Technology Co., Ltd., China), an LCR meter (E4980AL, Keysight), and a 3D‐printed bump. The TSA and 3D‐printed bump were attached to the side of each of the two sliders of the precision electric moving stage, and the two moved in opposite directions with the micro displacements. A TSA unit was connected to an LCR meter through a flexible print circuit (FPC) connector.

To test the distance detection capability and scanning characteristics of the TSA, it was attached to the underside of a 3D‐printed mold carrying weights. The mold was then placed on the surface of another 3D‐printed plate with bars. The system was towed by the slider of the moving stage using tapes, as shown in Figure S11 (Supporting Information).

For distance detection, pattern reconstruction, and Braille recognition experiments, the designed CMC consisting of three capacitance‐to‐digital converters was connected to the TSA.The real‐time capacitances of all sensing units were measured. A microcontroller (i.e., MCU) was employed to control the converters and transfer the data to the portable electronics.

## Conflict of Interest

The authors declare no conflict of interest.

## Supporting information

Supporting Information

Supplemental Video 1

Supplemental Video 2

Supplemental Video 3

Supplemental Video 4

## Data Availability

The data that support the findings of this study are available from the corresponding author upon reasonable request.
