# Supplementary material for: All‐Printed Finger‐Inspired Tactile Sensor Array for Microscale Texture Detection and 3D Reconstruction
Source: Adv Sci (Weinh). 2024 May 2;11(26):2400479. doi: 10.1002/advs.202400479 (PMC11234443; doi:10.1002/advs.202400479)
Supplement: Supplementary file 1 — Supporting Information [file ADVS-11-2400479-s001.pdf]

ADVANCED SCIENCE
Open Access

Supporting Information

for *Adv. Sci.*, DOI 10.1002/advs.202400479

All-Printed Finger-Inspired Tactile Sensor Array for Microscale Texture Detection and 3D Reconstruction

*Yilin Wang, Jiafeng Zhao, Xu Zeng, Jingwen Huang, Yading Wen, Juergen Brugger and Xiaosheng Zhang**

Supporting Information

All-printed finger-inspired tactile sensor array for microscale texture detection and 3D reconstruction

*Yilin Wang, Jiafeng Zhao, Xu Zeng, Jingwen Huang, Yading Wen, Juergen Brugger, and Xiaosheng Zhang**

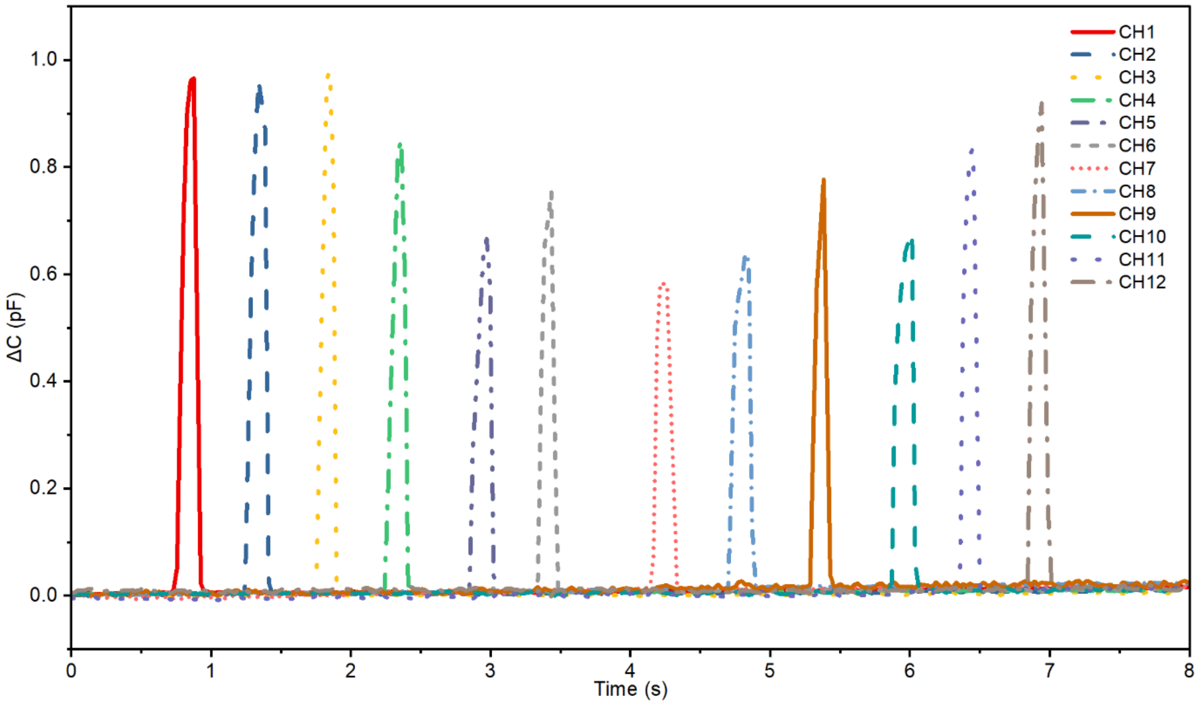

Figure S1. Response curves of 12 sensing units in a tactile sensor array. Each sensing unit was pressed with a finger one by one, and only the pressed unit responded. This proves that the signals of each sensing unit are highly isolated and will not be interfered by the signals from other units.

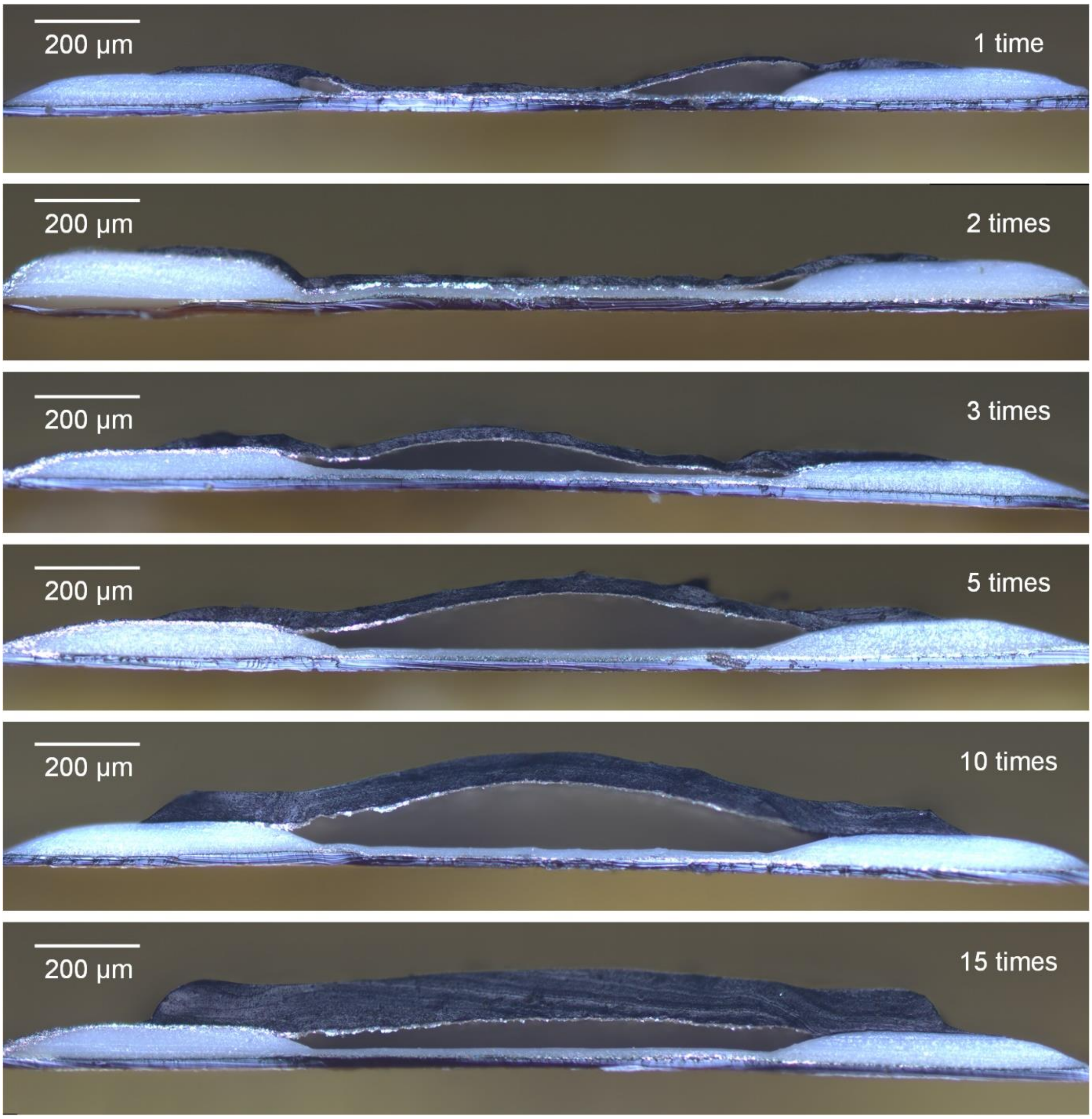

Figure S2. Microscope images of the cross-section of sensing units with different-times-printed beam layers. The beam layer's thickness increases with the printing time, and the beam layer will sag when the printing time is less than 5. In the process of releasing the sacrificial layer, the gas produced by the sublimation of sacrificial materials causes the beam to expand outward. In the range of 3 to 10 times, as the beam thickness increases, the sagging degree of the expanded beam decreases and thus the curvature becomes larger. When the number of prints reaches 15 times, the beam thickness is larger, so on the one hand, the degree of sagging is smaller, and on the other hand, the degree of expansion produced by the sublimation of sacrificial materials is smaller, and thus the curvature is lower than that in the case of 10 times.

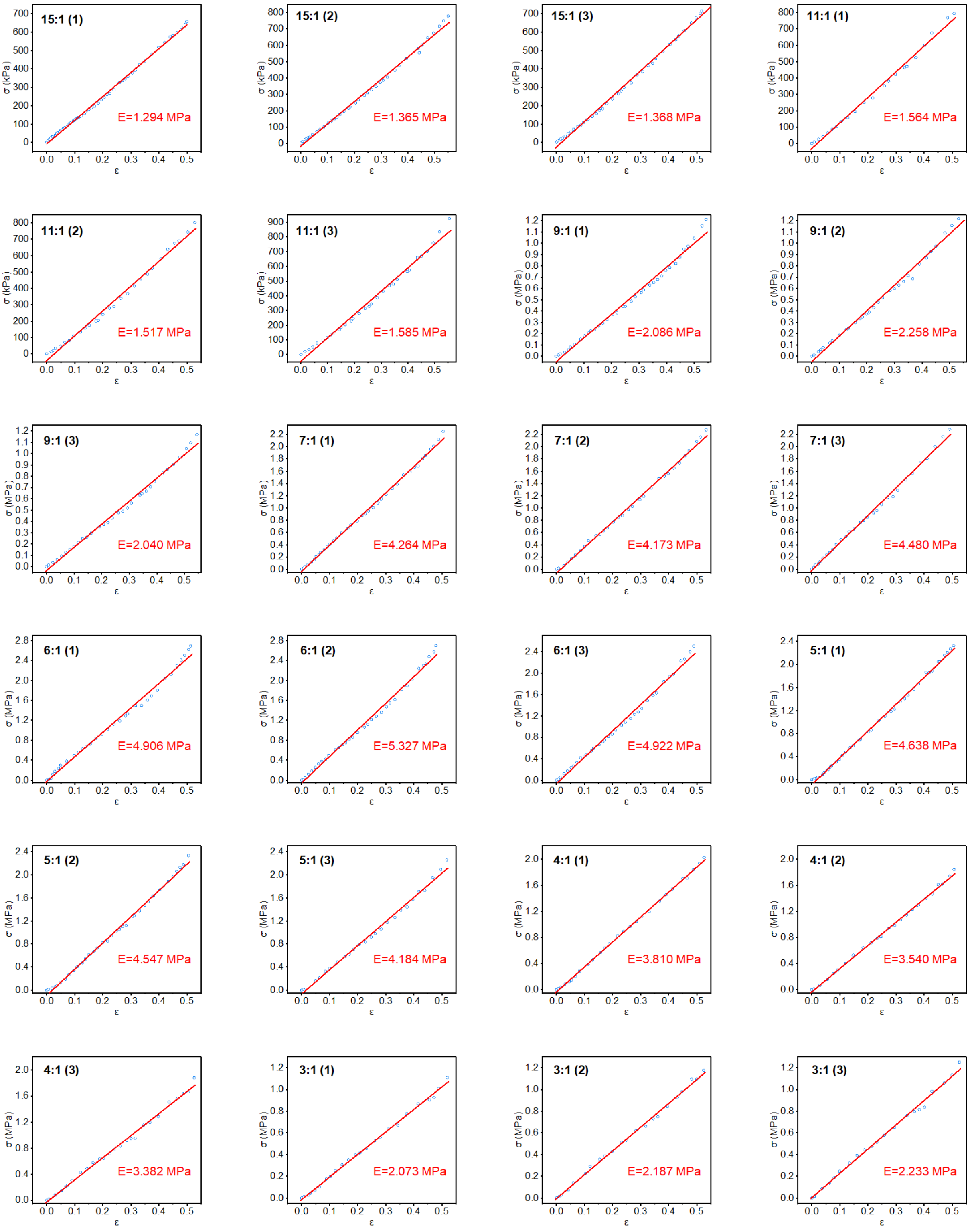

Figure S3. Variation of stress (σ) with strain (ε) for PDMS film with different ratios of base to curing agent (3:1, 4:1, 5:1, 6:1, 7:1, 9:1, 11:1, 15:1). Measurements were taken at three different points on each film. After each measurement, a linear fit was performed on the results, and the slope (E) of the fitted line is Young's modulus of the measured film.

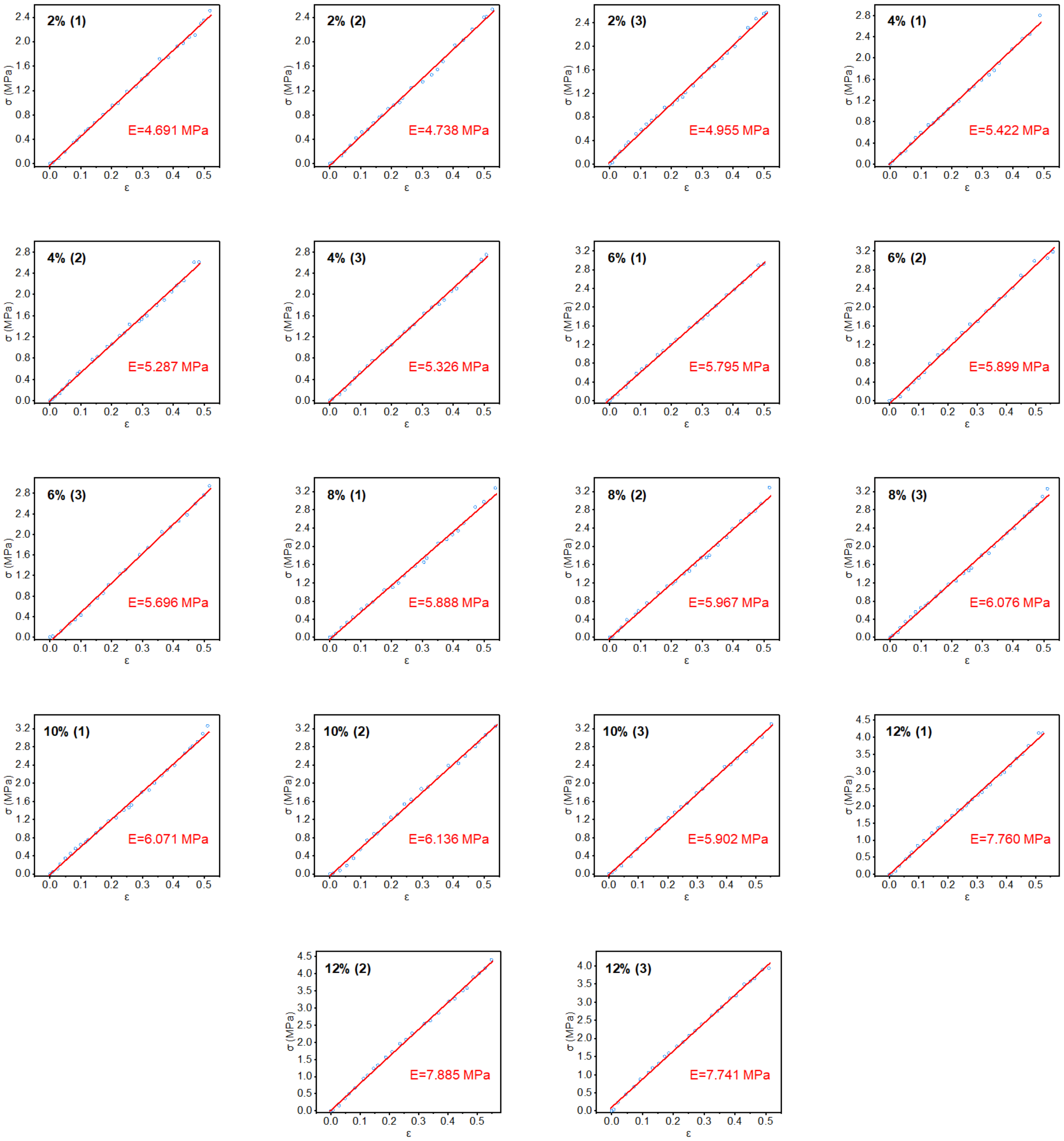

Figure S4. Variation of stress (σ) with strain (ε) for CB/PDMS film (ratio of PDMS base to curing agent is 5:1) with different weight percentages of carbon black. Measurements were taken at three different points on each film. After each measurement, a linear fit was performed on the results, and the slope (E) of the fitted line is Young's modulus of the measured film.

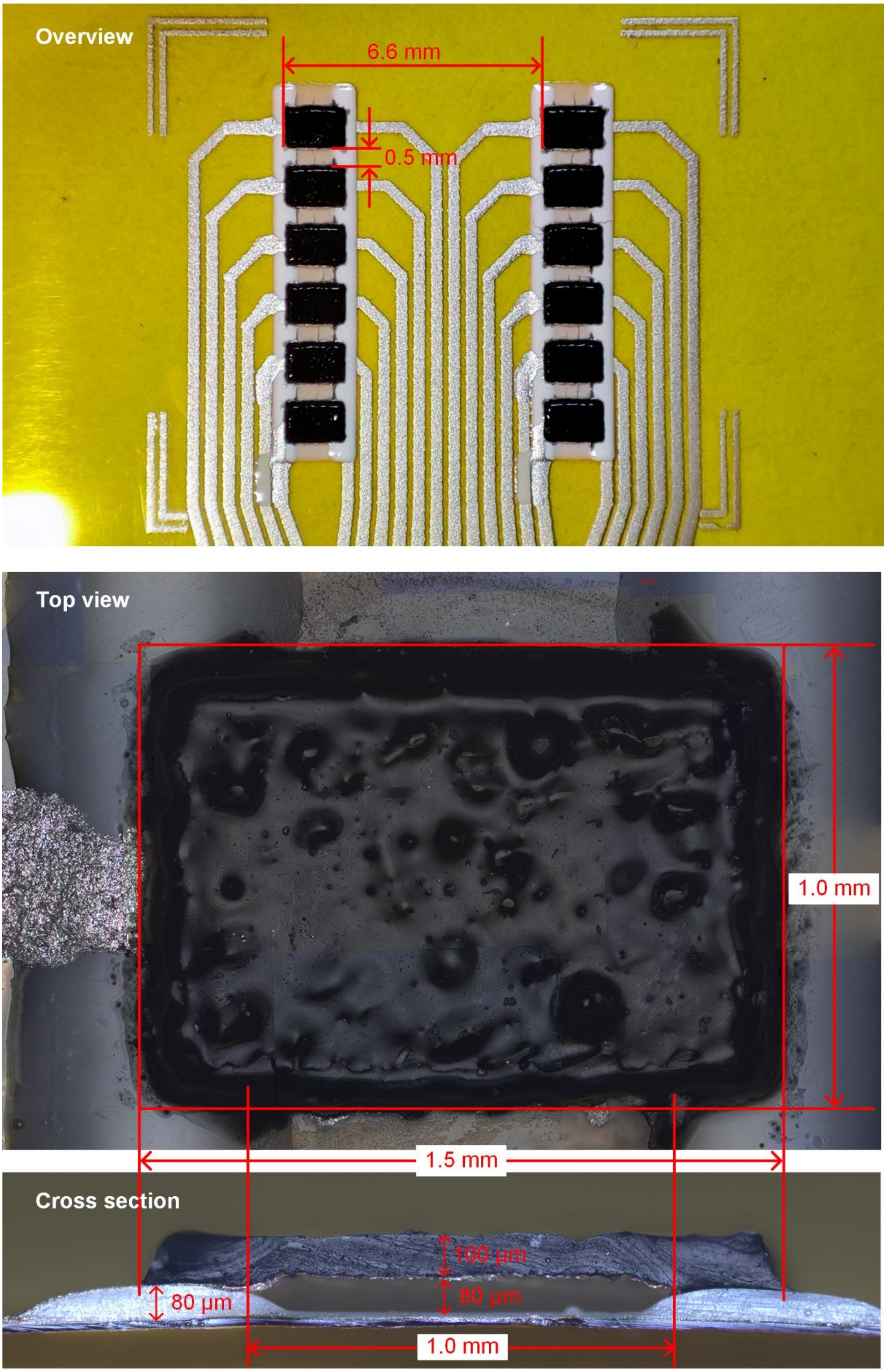

Figure S5. Photographs of the tactile sensor array and the sensing unit with size information.

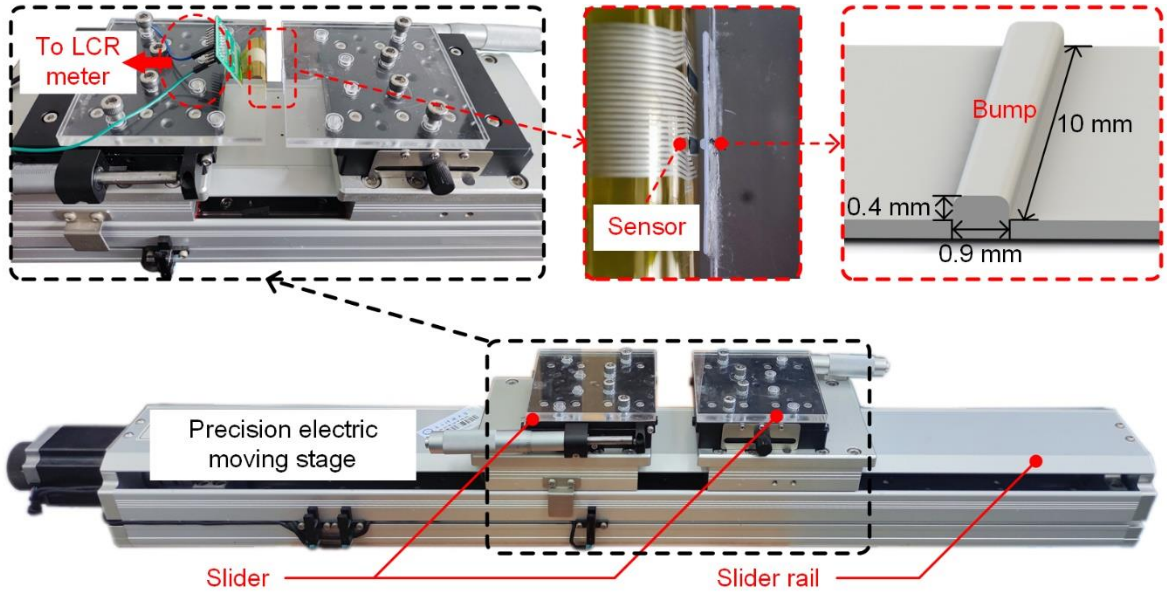

Figure S6. Customized testbench for characterization of the texture height detection ability of the sensing unit. The tactile sensor array and a 3D-printed bump were attached to the side of each of the precision electric moving stage's two sliders, and the two moved in opposite directions with micro displacements. One tactile sensor array sensing unit was connected to the LCR meter through a flexible print circuit (FPC) connector (green PCB). The size of the 3D-printed bump is 10 mm × 0.9 mm × 0.4 mm (length × width × height).

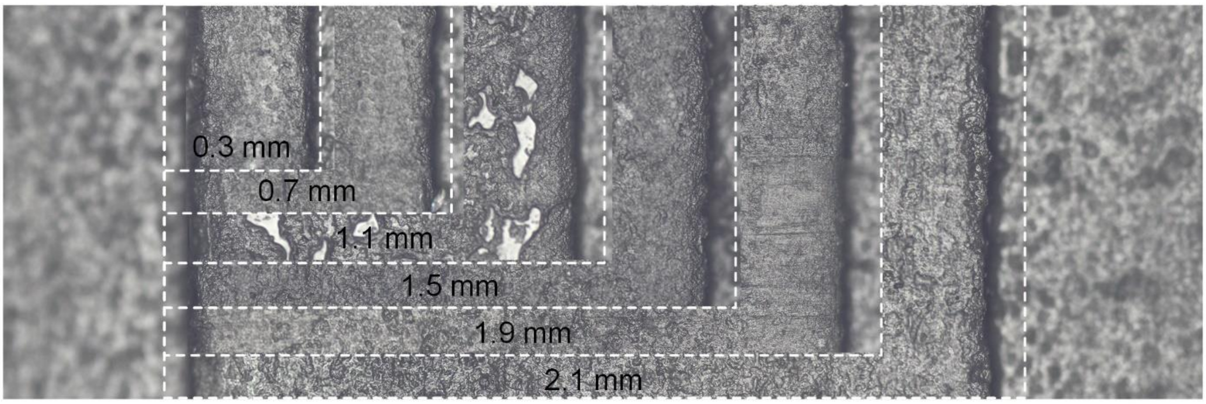

Figure S7. Microscopic photographs of 3D printed bars. These bars are all 100 μm in height and have different widths of 0.3 mm, 0.7 mm, 1.1 mm, 1.5 mm, 1.9 mm, and 2.1 mm.

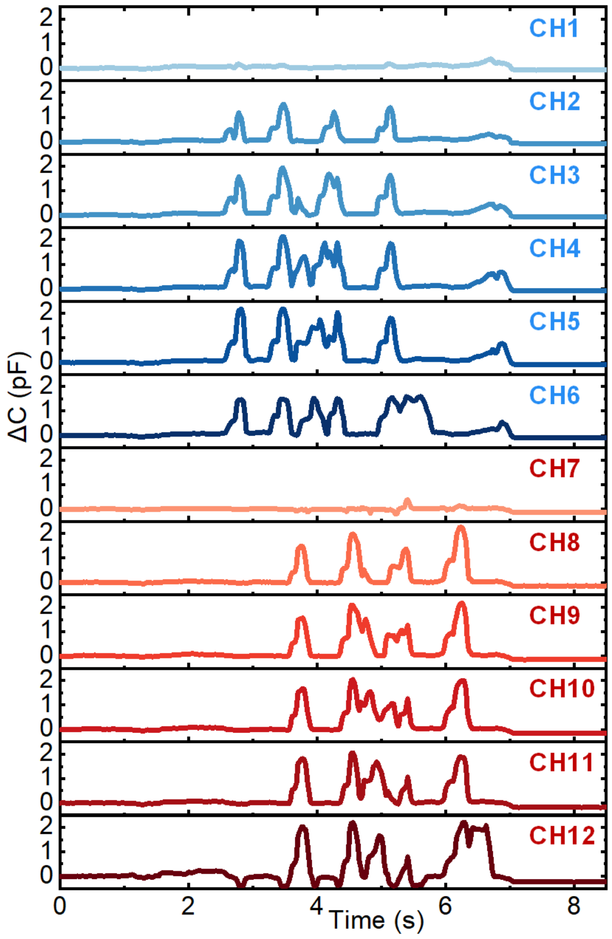

Figure S8. The response curves of each sensing unit when the tactile sensor array scans over an "IML" shaped surface pattern. CH1 to CH6 refer to the signals from the sensing units in the front column, and CH7 to CH12 refer to the signals from the sensing units in the rear column.

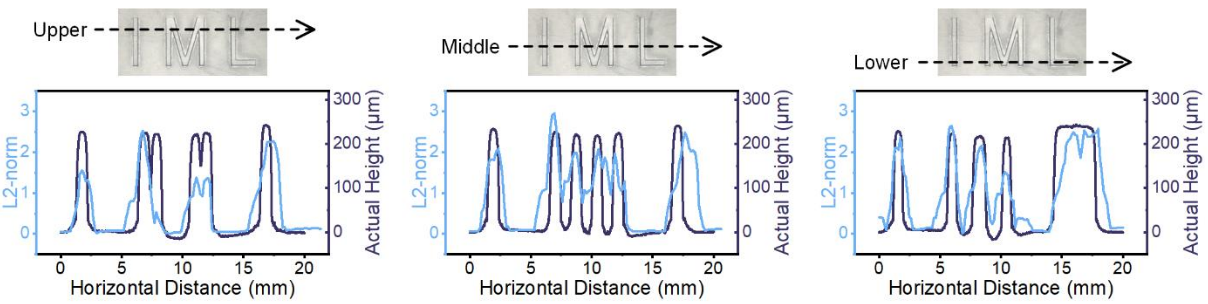

Figure S9. The height profiles of the actual structure on the upper, middle, and lower paths measured by a stylus profiler, and compared with the height profiles of the reconstructed model optimized by the L2-norm. There is a high degree of similarity between the model and the actual structure in detail.

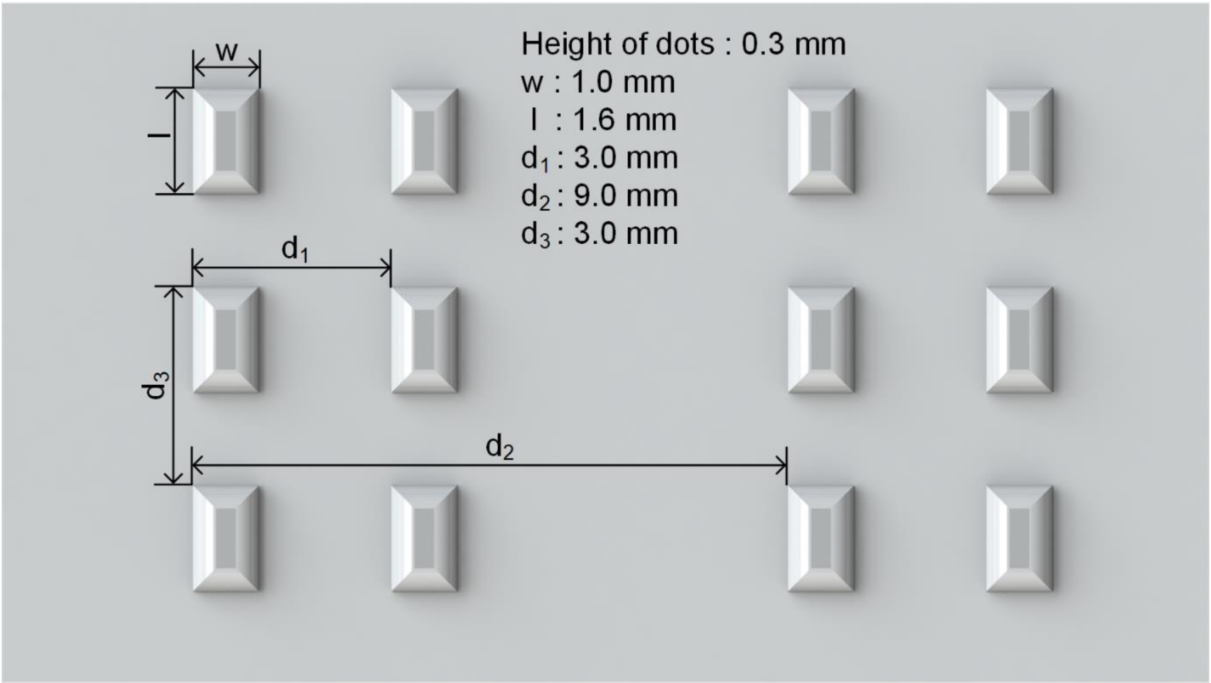

Figure S10. The dimension of braille characters consists of six braille dots.

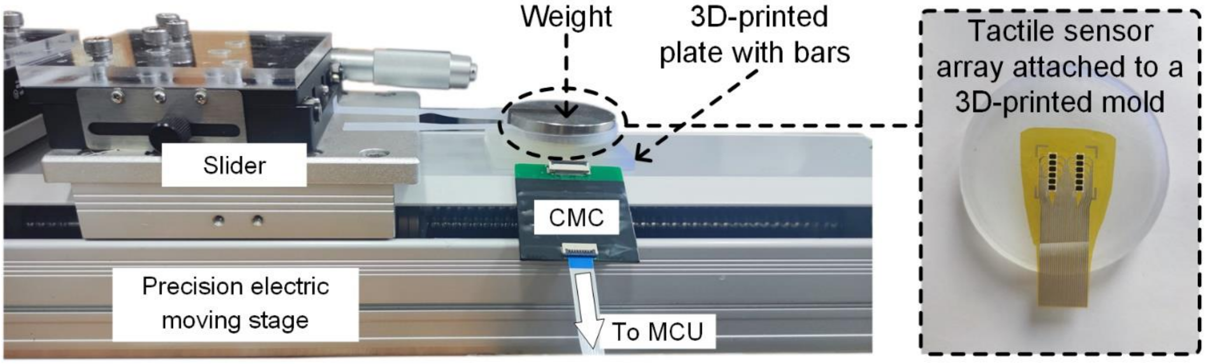

Figure S11. Customized testbench for characterization of the texture distance detection ability of the tactile sensor array. The tactile sensor array was attached to the bottom of a 3D-printed mold that carried 200g weights. The mold was attached to the surface of another 3D-printed plate with bars and pulled by the slider of the moving stage through tape.

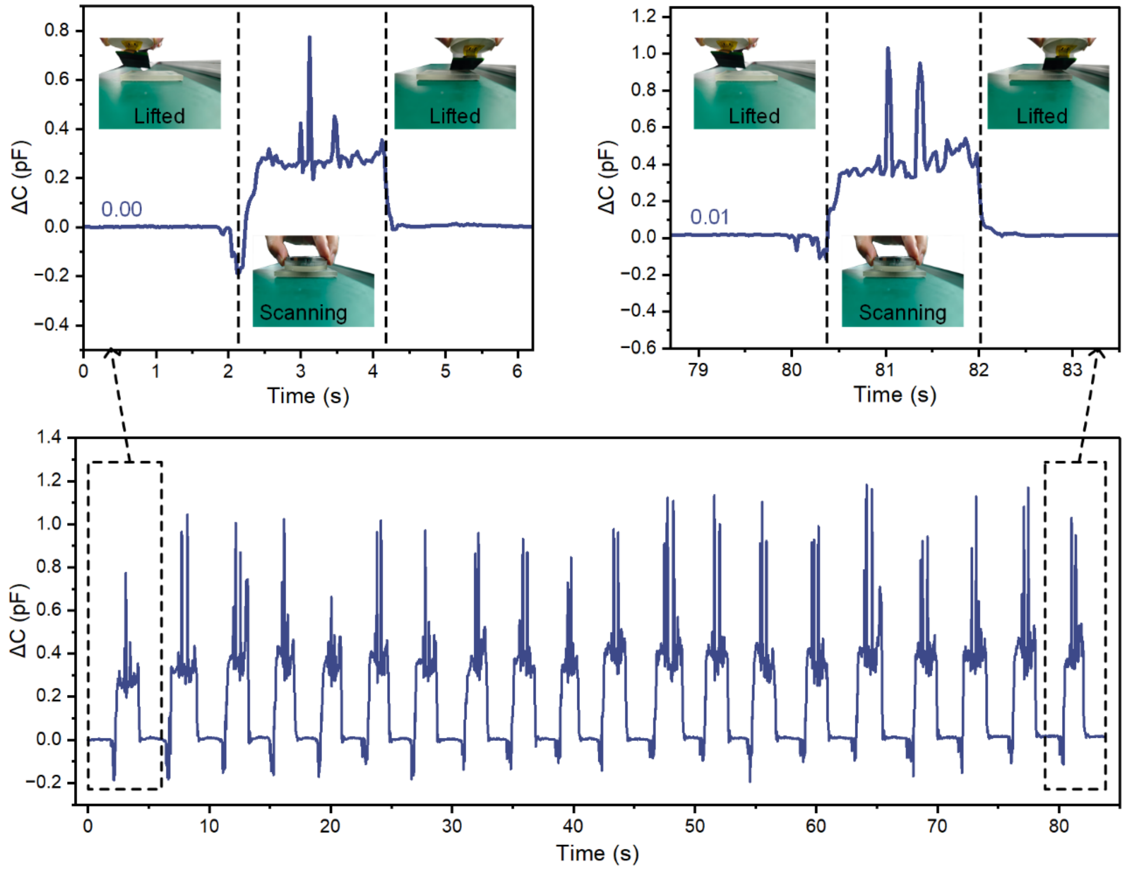

Figure S12. The baseline of ΔC exhibits good stability. The sensor array is lifted up before and after scanning to remain uncompressed. In order to easily control the position of the sensor, we use our hands rather than the machine to drive the sensor, so there will be unexpected fluctuations in the signal, nevertheless, the focus is on the baseline of the signal. After 20 cycles, the baseline of the ΔC only changes from 0 to 0.01, which is negligible. This means that when the external force applied to the sensor is released, the capacitance value of the sensing unit will restore to the initial state, so when the sensor undergoes the next scanning, the initial capacitance value will be the same as that of the previous scanning, and it will not have any effect on the reconstruction results.

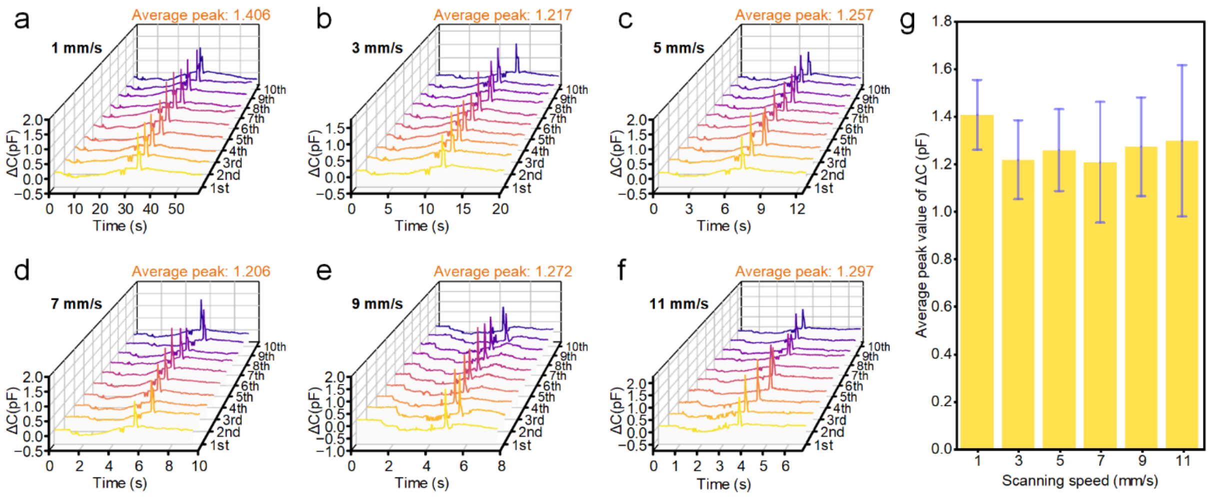

Figure S13. Peak value evaluation of the response curves during scanning. a)-f) Response curves of the sensing unit when the TSA scans over a 500 μm wide and 100 μm high bar with different scanning speeds. g) The average peak values of the response curves. Even at the same scanning speed, the peak ΔC fluctuates considerably, whereas the average value of the peak ΔC at different speeds is relatively close to each other, at around 1.25 pF. It indicates that the scanning speed is not the cause of the fluctuating peak value of ΔC, but the instability of the displacement system in the testbench. The sensor and the loaded mold are towed by the slider of the moving stage via tapes to achieve movement. However, tape connections are not rigid, due to friction, the TSA wobbles as it moves, which causes the sensor units to contact the bars at different angles, resulting in different capacitance changes. Moreover, the faster the TSA moves, the more pronounced this fluctuation will be, resulting in a larger error.

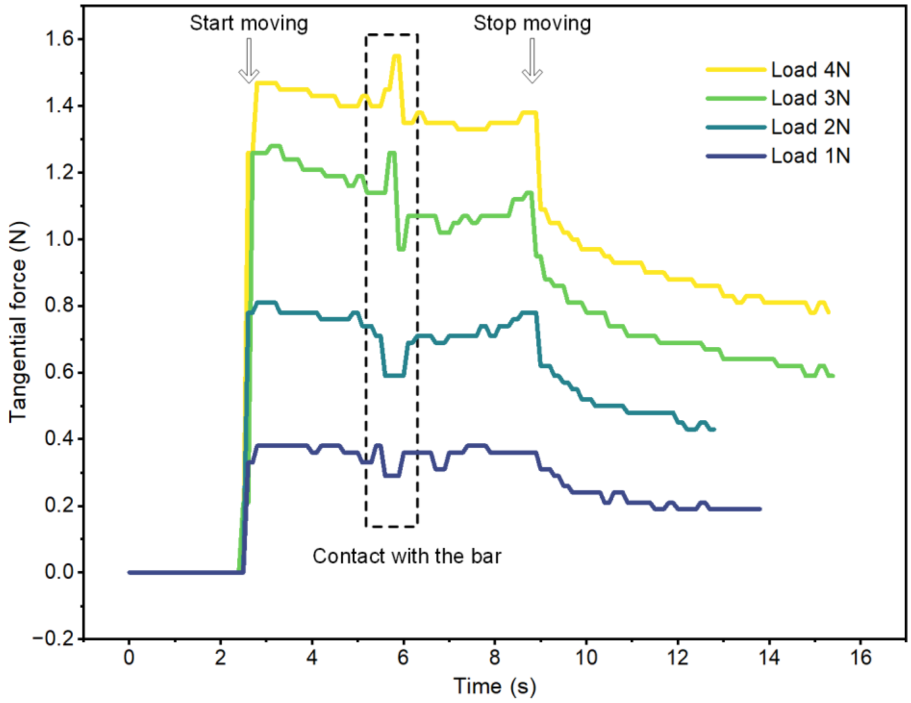

Figure S14. Curves of tangential force variation as the sensor scans the 500 μm wide and 100 μm high bar under different loads. The tangential force increases as the load increases, and changes dramatically when the sensor comes into contact with the texture.
